# Degradation of Some Polymeric Materials of Bioreactors for Growing Algae

**DOI:** 10.3390/ma19020384

**Published:** 2026-01-18

**Authors:** Ewa Borucińska-Parfieniuk, Ewa Górecka, Jakub Markiewicz, Urszula Błaszczak, Krzysztof J. Kurzydlowski, Izabela B. Zglobicka

**Affiliations:** 1 Faculty of Mechanical Engineering, Bialystok University of Technology, 45C Wiejska Str., 15-351 Bialystok, Poland; k.kurzydlowski@pb.edu.pl; 2 Instytut Nauk o Morzu i Środowisku, Uniwersytet Szczeciński, 16A Mickiewicza Str., 70-383 Szczecin, Poland; 3 Faculty of Electrical Engineering, Bialystok University of Technology, 45C Wiejska Str., 15-351 Bialystok, Polandu.blaszczak@pb.edu.pl (U.B.)

**Keywords:** transparent polymers, photodegradation, optical transmittance, algal bioreactors, bioreactors

## Abstract

**Highlights:**

**What are the main findings?**

- PMMA and PMMA_R_ exhibited the highest light transmission and surface stability.
- PC and PET showed moderate degradation, while PC_2UV_ suffered severe optical losses.
- Vapor-exposed zones were more prone to degradation than fully immersed ones.

**What are the implications of the main findings?**

- Both types of PMMA are recommended for long-term use in bioreactors.
- Reduced transmittance lowers cultivation intensity and should be minimized in bioreactor use.
- Contact of the material with the vapor inside the bioreactor should be as little as possible.

**Abstract:**

Transparent polymeric materials such as poly(methyl methacrylate) (PMMA), polycarbonate (PC), and polyethylene terephthalate (PET) are widely used as glass alternatives in algal bioreactors, where optical clarity and mechanical stability are crucial. However, their long-term use is limited by surface degradation processes. Photodegradation, hydrolysis, and biofilm accumulation can reduce light transmission in the 400–700 nm range essential for photosynthesis. This study examined the aging of PMMA, PC, and PET under bioreactor conditions. Samples were exposed for 70 days to illumination, culture medium, and aquatic environments. Changes in their optical transmittance, surface roughness, and wettability were analyzed. All polymers exhibited measurable surface degradation, characterized by an average 15% loss in transparency, significant increases in surface roughness, and reduced contact angles. PMMA demonstrated the highest optical stability, maintaining strong transmission in key blue and red spectral regions, while PET performed the worst, showing low initial clarity and the steepest decline. The most severe surface degradation occurred in areas exposed to the receding liquid interface, highlighting the need for targeted cleaning and/or a reduction in the size of the liquid–vapor transition zone. Overall, the results identify PMMA and recycled PMMA (PMMA_R_) as durable, cost-effective materials for transparent bioreactor walls.

## 1. Introduction

Polymer materials, owing to their versatility, low density, and ease of processing, play a key role in numerous industrial and everyday applications [1,2,3]. Transparent plastics, such as poly(methyl methacrylate) (PMMA), polycarbonate (PC), and polyethylene terephthalate (PET), are increasingly replacing glass because they combine optical clarity with acceptable mechanical properties and higher toughness [2,4]. Despite these advantages, their relatively low hardness, scratch resistance, and susceptibility to environmental degradation remain important limitations [2,5].

Polymer degradation is an irreversible process involving the breakdown of covalent bonds in molecular chains. It lowers molecular weight and progressively deteriorates the material’s physical, mechanical, and optical properties [5]. Among the most severe degradation pathways are photobiodegradation and hydrolytic or microbiologically induced changes, particularly under conditions combining light exposure, humidity, and the presence of microorganisms [6,7,8].

These processes are highly relevant for bioreactors used in algal cultivation, which are constructed from light-transmitting polymers [9].

Such reactors operate under intense solar radiation and are in constant contact with culture media of variable physicochemical composition. The transparent walls, usually made of PMMA, PET or PC, are critical to both structural integrity and efficient light transmission [10,11]. However, their durability is compromised by yellowing and reduced transmittance due to photodegradation, surface alterations caused by microbial activity, and biofilm accumulation that further restricts light transmission, ultimately lowering algal productivity [12].

For an informed choice of materials, transparent polymers differ in chemistry, properties, and cannot be treated as a uniform material class. Their long-term performance must be assessed by testing their optical, mechanical, and surface properties under conditions that mimic real operating environments.

PMMA is an amorphous, transparent polymer with excellent optical performance, exhibiting light transmittance of approximately 92% [13,14]. It is easy to process and relatively inexpensive. However, its low resistance to UV radiation, poor scratch resistance, and limited durability in seawater significantly restrict its use in demanding environments [2,15]. Exposure to these factors results in swelling, yellowing, and the formation of microcracks, leading to pronounced deterioration of both mechanical and optical properties [16].

PC offers light transmittance in the range of 88–90% [17] and superior mechanical resistance compared to PMMA. Nevertheless, it undergoes hydrolytic degradation under aqueous conditions, particularly in the presence of salt and UV radiation. This process induces surface damage such as pitting and microcracks, accompanied by a progressive loss of transparency [18,19].

PET combines good chemical resistance with favorable barrier properties, while its optical transmittance typically ranges between 85 and 90% [20]. Despite these advantages, PET also undergoes slow hydrolysis in marine environments, especially under UV exposure, leading to a decline in physicochemical stability and increased brittleness [6].

Although transparent polymers are increasingly employed in water-contact applications [11], systematic long-term studies on their aging and degradation under combined exposure to microorganisms and UV light remain scarce.

The present study addresses this gap by evaluating the aging and degradation behavior of three widely used transparent polymers—PMMA, PC, and PET—selected for their favorable optical properties and broad commercial availability [21,22,23]. Specifically, the effects of photodegradation, culture medium, and aquatic exposure on their optical and surface characteristics were investigated. Experimental analyses included measurements of light transmission, surface roughness, and wettability, providing a comprehensive assessment of material changes under conditions simulating real operational environments. The results are discussed in the context of defining practical material selections and in-service procedures.

## 2. Materials and Methods

### 2.1. Materials

Materials investigated in this study included commercially available sheets of the following: PC, PC_2UV_ (polycarbonate with double UV layer), PET, PMMA, and PMMA_R_ (recycled polymethylmethacrylate). According to the Safety Data Sheets, the light transmittance of each of these materials specified by manufacturers was at least 88%. Key properties of the studied materials, based on manufacturer specifications, are listed in Table 1. Manufacturer-reported transmittance values were taken from the Safety Data Sheets; however, these documents do not specify the wavelength range or the instrumentation and measurement geometry used. Consequently, the manufacturer values cannot be directly compared with the measured spectral transmittance.

### 2.2. Sample Preparation

**Figure 1:**
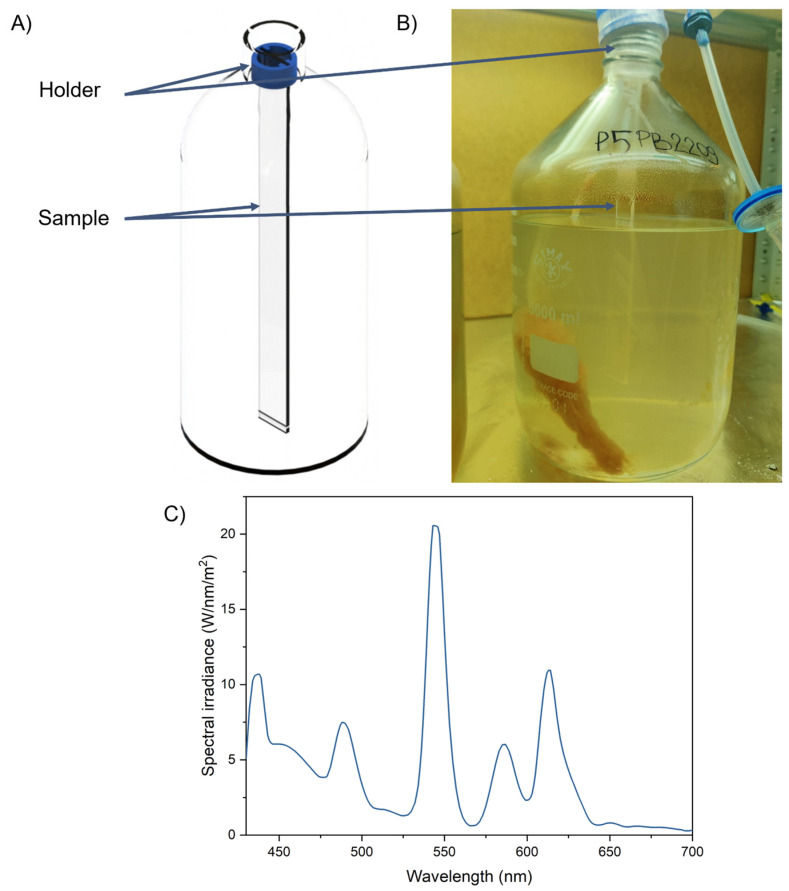
Explanation of the sample immersion: (**A**) position of the sample; (**B**) photo of bioreactor in the form of a carboy with placed sample, made on the last day of experiment; (**C**) spectral distribution of the LED used for cultivation in the bioreactor.

Flat specimens with dimensions of 25 × 290 × 3 mm were cut from commercially available polymer sheets (PMMA, PMMA_R_, PC, PC_2UV_, and PET) with one specimen obtained from each sheet and inspected for surface defects. The specimens were then mounted inside the bioreactors as illustrated in Figure 1A,B.

### 2.3. Bioreactor Setup and Culture Conditions

Carboy-type glass bioreactors with a total volume of 5 dm^3^ were used (Figure 1B). Non-axenic cultures of the marine diatom *Thalassiosira pseudonana* isolated from the Baltic Sea, in Niechorze, Poland, on 9 June 2022 were maintained for 70 days under controlled conditions. The temperature was kept at 22 ± 1 °C, and illumination was provided by LED lamps (luminous flux of 6500 lm) corresponding to light intensity of 80 μmol photons m^−2^ s^−1^. The photon flux density was measured at the bottom of the bioreactor, 60 cm from the light source. Prior to the inoculation with *T. pseudonana,* all bioreactor components (bottles, caps, and tubing) as well as the distilled water and salt were autoclaved in 121 °C for 20 min. Nutrient, trace metal, and vitamin stock solutions were added aseptically through 0.2 µm sterile syringe filters. Each bioreactor was inoculated with 3 mL of *T. pseudonana* culture using a sterile plastic pipette. The cap–bottle interface was sealed with Parafilm, and aeration was supplied through 0.2 µm sterile air filters. The 70-day exposure time was chosen to capture long-term effects of continuous contact with the culture medium and microalgae on the specimens, while minimizing the risk of unintended bacterial contamination during prolonged cultivation. The spectral distribution of the light source is shown in Figure 1C. The culture medium consisted of f/2 medium prepared with distilled water [24], with salinity adjusted to 20‰ using Aquaforest Reef Salt. The pH was approximately 8, as determined using Macherey-Nagel pH test strips. Aeration was supplied at the bottom of each reactor using atmospheric air to ensure mixing and gas exchange.

### 2.4. Post-Exposure Handling and Analysis

After 70 days of exposure, the specimens were carefully removed from the bioreactors and examined in “as-exposed” condition. Thereafter, the specimens were cleaned in accordance with standard bioreactor maintenance procedures. The surfaces were moistened with water. Deposits and residues were gently removed using a soft sponge and a mild surfactant commonly employed for bioreactor cleaning. The cleaning solution used contained the following main ingredients: 15–30% anionic surfactants, 5–15% nonionic surfactants, methylisothiazolinone, phenoxyethanol, and fragrances. Finally, the specimens were rinsed with distilled water to remove any remaining impurities and dried under ambient conditions. This cleaning procedure corresponds to that typically used by bioreactor operators. Following cleaning, the specimens were subjected to further investigation.

**Figure 2:**
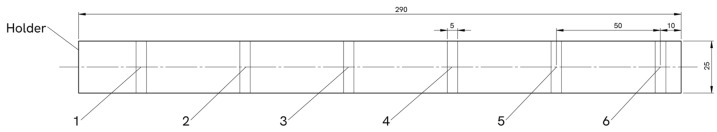
Designation of the observation fields on the specimens removed from bioreactors.

Surface degradation of the specimens removed from the bioreactors was investigated in six zones defined along their length, as shown in Figure 2. These observation fields were selected to represent different exposure conditions:

- To humid air inside the reactor (zones 1, 2);
- To variable conditions—transition zone: air–water (zone 3);
- To direct contact with the culture medium (zones 4, 5, 6).

Because the culture medium level gradually descended during operation, the transition zone (zone 3) was intermittently exposed to both air and liquid.

### 2.5. Methods

Spectral transmission of the samples was measured using a Stellarnet spectrometer (StellarNet, Inc., Tampa, FL, USA). The setup consisted of a tungsten-halogen lamp with a fiber-optic output coupled to a fiber-optic spectrometer. Transmitted light was recorded over the wavelength range of 400–700 nm with a step size of 0.5 nm, and all results were normalized to the transmission of air. In each observation zone (see Figure 2), five measurements were taken in five different locations. The spectra and points on the graphs presented are the results of the arithmetic mean of the measurements presented.

Surface wettability was assessed using an Ossila goniometer (Ossila Ltd., Sheffield, UK) with dedicated analysis software. Distilled water droplets of 15 μL were deposited on the sample surface, and images were captured 10 s after deposition. Five droplets were measured in each observation zone at various locations, as shown in Figure 2. The work of adhesion (W_A_) was calculated from the contact angle (θ) using the Young–Dupré relationship:

(1) $$W_{A}=\gamma_{LV}\left(1+cos\theta \right),$$

where $\gamma_{LV}$ is the surface tension of the test liquid.

Results are presented as arithmetic means, and error bars represent the standard deviation.

Surface roughness was evaluated using a Mitutoyo Surftest SJ-500 profilometer (Mitutoyo, Kawasaki, Japan) equipped with a 1 μm radius stylus. The stylus traversed the sample at 0.2 mm × s^−1^ over a vertical range of 8 μm. Profiles were recorded along lengths of 0.08 mm, 0.25 mm, and 0.80 mm, and R_a_ values were calculated. As with the other measurements, the roughness of all five samples was measured at the six locations indicated in Figure 2. The arithmetic mean of ten measurements was reported for each observation area; error bars represent the standard deviation.

## 3. Results

### 3.1. Light Transmission of Virgin Samples

**Figure 3:**
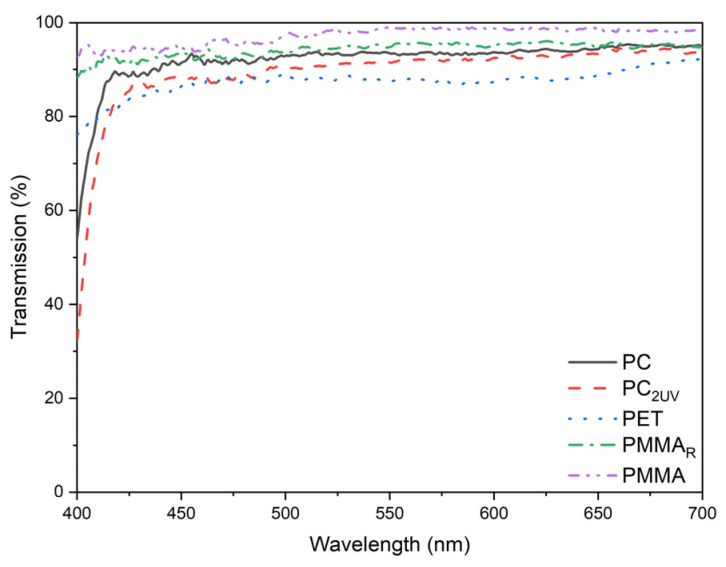
Spectral transmission of virgin polymer samples (PMMA, PMMA_R_, PC, PC_2UV_, PET) in the 400–700 nm range prior to exposure in bioreactors.

The measured light transmission media of the virgin polymer samples in the 400–700 nm range is shown in Figure 3. Transmission was relatively uniform across most of the range, with notable differences only in the 400–425 nm region. PMMA exhibited nearly constant transmission above 97%, while PET increased from 75% to 85%, PC from 30% to 85%, and PC_2UV_ from 55% to 85%. These values are consistent with manufacturer specifications and suggest that, in terms of light transmission, PMMA is particularly suitable for these algae species which absorb light in the 400–425 nm range.

### 3.2. Light Transmission After Exposure

**Figure 4:**
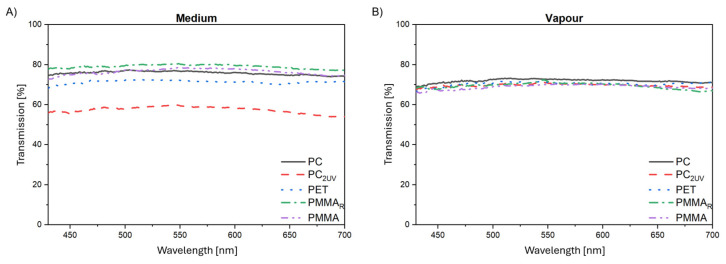
Transmission spectra of polymer specimens after 70 days in bioreactor: (**A**) fully immersed in culture medium, (**B**) exposed to vapor above the medium.

**Figure 5:**
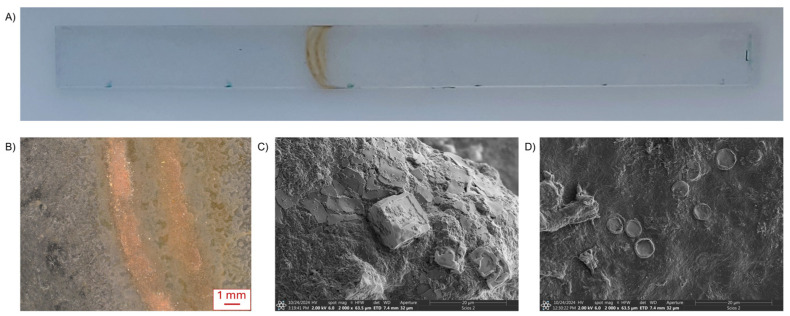
(**A**) Photo of PC sample after exposure. (**B**) Light microscopy image of deposit on PC sample. SEM images of deposit on (**C**) PMMA and (**D**) PET sample.

Transmission spectra of specimens exposed, without cleaning, to vapor-above-medium conditions are shown in Figure 4, whereas microscopy images of these zones are presented in Figure 5.

Figure 5A shows the typical appearance of a sample after exposure to bioreactor conditions. The most visible feature to the naked eye is the sediment from the transition zone, observed on all tested samples and differing mainly in color intensity. The light microscopy image (Figure 5B) indicates that deposition is not limited to a “coffee-ring” pattern, as sediment is also present outside the visibly streaked area. Further analysis of material collected from the sediment revealed distinct microscale components. Scanning electron microscopy shows crystallized particles originating from the microalgae growth medium (Figure 5C), alongside intact diatom frustules embedded within the deposited matrix (Figure 5D). Taken together, these observations indicate that the sediment layer in the transition zone comprises both inorganic constituents of the medium and biological material.

In general, biofilm on submerged samples typically contains live and dead algal cells, heterotrophic bacteria, and an extracellular polymeric substance (EPS) matrix composed mainly of polysaccharides, proteins, lipids, and nucleic acids [25,26,27]. Deposits above the liquid line (the so-called transition zone) are of particular interest because of whitish or translucent residue bands formed as a result of evaporation and condensation cycles [28]. Dissolved salts and organics are transported upward via aerosolized microdroplets and capillary wicking.

**Figure 6:**
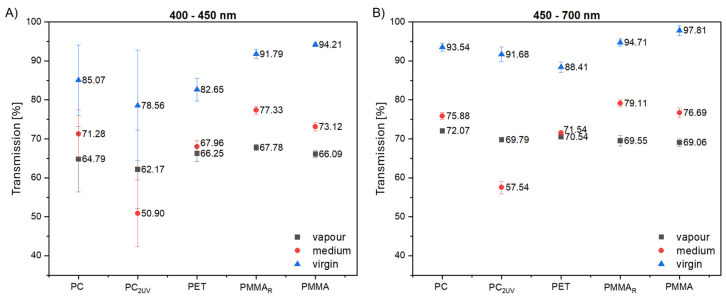
Comparison of mean transmission values for vapor-exposed, fully immersed, and virgin polymer samples: (**A**) in range 400–450 nm; (**B**) in range 450–700 nm. Legend: vapor—sample above the medium; medium—sample immersed in the liquid; virgin—sample before any treatment.

All materials investigated exhibit measurable surface degradation, with reductions in light transmission on average of approximately 10% for PC and PET, 20% for PMMA and PMMA_R_, and up to 30% for PC_2UV_, for wavelengths below 450 nm. Figure 6 summarizes mean transmission values for the vapor-exposed, fully immersed (designated as medium), and virgin samples. Notably, most materials showed lower transmission in vapor-exposed zones, whereas PC_2UV_ exhibited more pronounced degradation in immersed regions.

A drop in culture level caused by, i.e., evaporation, harvesting or tilting, might result in a visible ring/band [29,30]. The coffee-ring effect was observed during our investigations (Figure 5). The geometry of the ring might be modulated by microorganisms and their secreted biopolymers; the net outcome is typically an enhanced rim deposit [30].

Based on the transmission measurements, PMMA and PC were selected for more detailed analysis. PMMA demonstrated the highest optical stability, while PC was chosen for its suitability in the composition. Subsequent investigations focus on these materials, including measurements of post-cleaning transmission, surface roughness, and work of adhesion calculations.

### 3.3. Light Transmission After Cleaning

Light transmission was also investigated on post-cleaning samples. After 70 days of bioreactor exposure, the specimens were collected, cleaned to remove residual medium and biofilm, and air-dried. Transmission values for cleaned samples are designated as “ac”.

**Figure 7:**
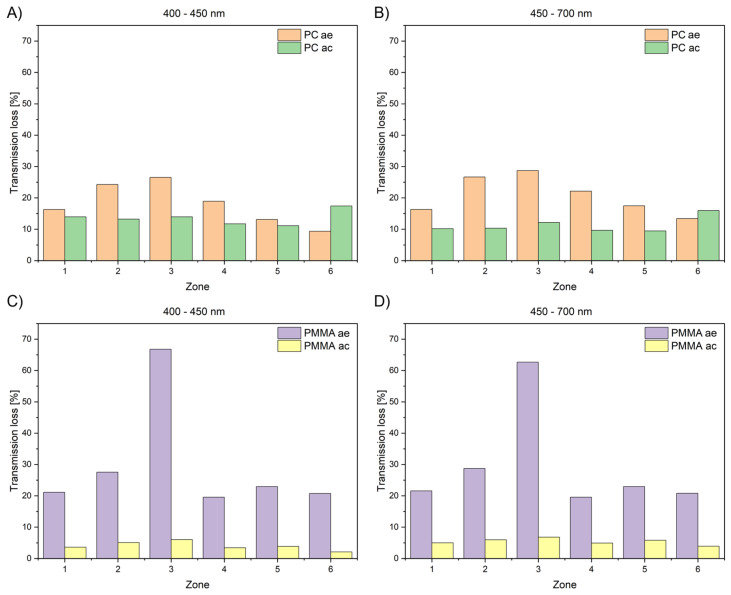
Mean transmission of PMMA and PC specimens measured in different sections relative to virgin sample: ae—after exposure; ac—after cleaning. (**A**) PC in range 400–450 nm; (**B**) PC in range 450–700 nm; (**C**) PMMA in range 400–450 nm; (**D**) PMMA in range 450–700 nm.

Figure 7 compares average light transmission of PMMA and PC after exposure (ae) and after cleaning (ac). It can be observed that the cleaning procedure (as described in Section 2.4) brought about a significant increase in the transmission values. For PMMA samples, this increase exceeded 15%. In comparison to the virgin-state samples, PC showed significant loss of light transmission post-cleaning (ca. 15% at 400–450 nm and ca. 10% at 450–700 nm), whereas PMMA nearly regained its range of values for virgin samples (5% lower across both ranges).

**Figure 8:**
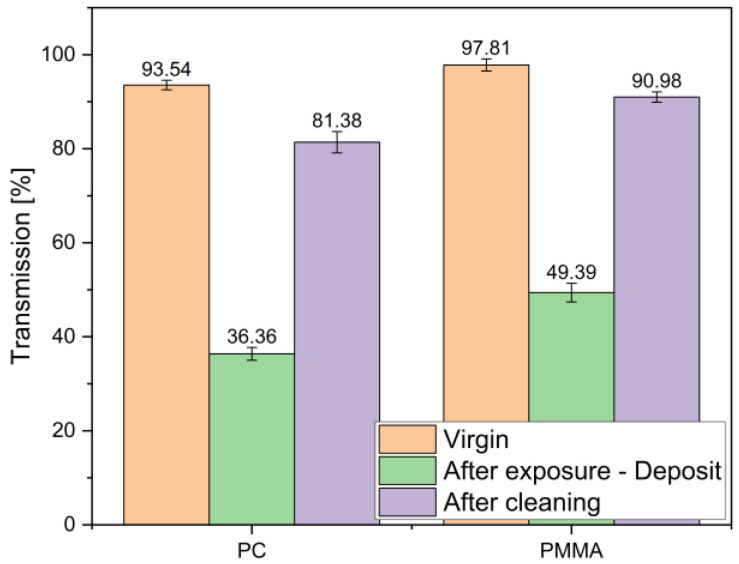
Effect of deposits on light transmission (450–700 nm) in PMMA and PC. Measurements performed through receding liquid zones after exposure compared with virgin material.

After exposure (before cleaning), transmission losses were highest in areas affected by receding liquid, likely due to biofilm deposits (Figure 8).

Generally, transmission losses were highest in areas of receding culture liquid. For PC/PMMA after exposure, light transmission was 36.36/49.39%, respectively. The applied cleaning operation resulted in an increase to 81.38/90.98%, respectively. This implies that in such an area, deposited biofilm resists cleaning. This is likely to be related to longer time of deposit resting and accumulation of floating species, both organic and inorganic, on the surface of liquid.

### 3.4. Surface Roughness

**Figure 9:**
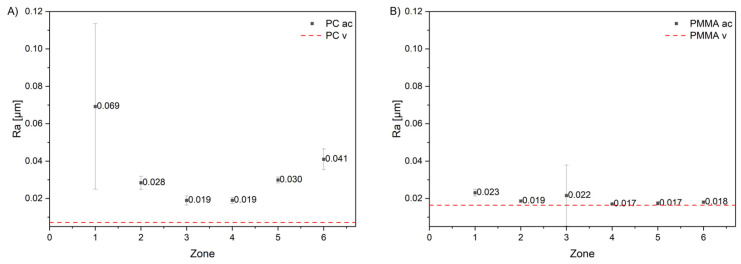
Change in surface roughness (R_a_) relative to virgin sample of (**A**) PC and (**B**) PMMA. Legend: ac—after cleaning, v—virgin. Zone 3 corresponds to areas exposed to receding liquid.

Results of surface roughness (R_a_) measurements are presented in Figure 9 and Table 2. It can be noted that cleaning of PC samples only partially reduced the roughness, particularly in zone 3. In contrast, PMMA largely recovered to its original roughness, indicating better surface stability and lower susceptibility to permanent mechanical damage. In the case of PC, surface roughness increased significantly in the first zone, located above the culture medium, and least in the transition zone. The increase in roughness in the medium-contact zone was comparable to that observed above the medium. The situation is different for PMMA. After cleaning, the material practically returned to its pre-exposure roughness. A noticeable increase in Ra was observed only in the transition zone. This zone is also characterized by a large deviation, indicating considerable heterogeneity. In the vapor zone, R_a_ increased slightly but visibly, suggesting stronger surface interactions compared to the zone in constant contact with the medium.

### 3.5. Wettability and Work of Adhesion Calculations

**Figure 10:**
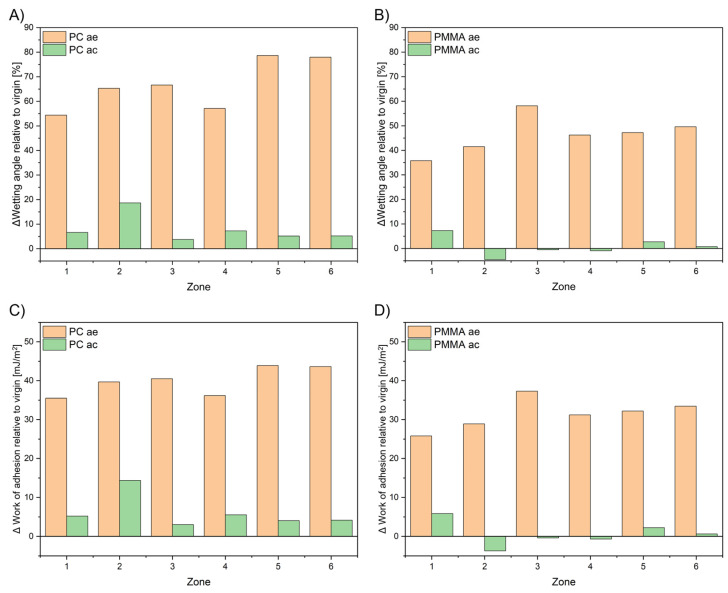
Changes relative to virgin materials across exposure zones: (**A**) wetting angle of PC, (**B**) wetting angle of PMMA, (**C**) work of adhesion of PC, and (**D**) work of adhesion of PMMA. Values indicate increased hydrophilicity after exposure, particularly in submerged zones.

Values of wetting angle as well as calculated values of work of adhesion calculations are plotted in Figure 10. Experimental values of wetting angle indicate increased hydrophilicity for tested materials after exposure. In terms of work of adhesion calculations, all values increase by ca. 35% in comparison to the virgin sample. The cleaning operation reduced work of adhesion for PC but did not fully restore virgin values. It suggests incomplete biofilm removal or permanent surface alteration. PMMA generally regained original work of adhesion, except for a slight increase in zone 1, consistent with roughness observations. It should be noted that the vapor zone, located above the liquid surface in the bioreactor, represents a particularly aggressive environment for polymeric material. Surface in this region is cyclically exposed to humid air, condensate droplets, and illumination, leading to alternating wetting and drying. These conditions promote localized accumulation of biofilm and inorganic deposits, enhance oxidative and hydrolytic reactions, and accelerate surface roughening. As a result, higher work of adhesion in zone 1 has been observed, with reduced optical clarity and more advanced aging compared to fully immersed regions.

## 4. Discussion

Over the past several years, research on polymer–light interactions have expanded considerably, driven by the need to understand both beneficial and detrimental effects on material performance in diverse applications. Ultraviolet (UV) radiation, with its higher energy compared to visible light, can readily induce polymer degradation [31,32]. Moisture can accelerate chain crystallization or hydrolytic degradation, altering critical properties such as refractive index [33,34].

Closed and semi-closed photobioreactors commonly experience wall biofouling. Microalgal cells adhere to the inner walls and are embedded in an extracellular polymeric substances (EPS) matrix, often alongside associated bacteria. The developing biofilms reduce through the wall light transmission. Their formation might be reversible and/or irreversible, depending on the species, light, and materials used for construction of bioreactor [25,35].

In this study, using witness samples, we systematically evaluated effects of surface degradation and biofilm formation for five commonly used transparent polymers—PMMA, PMMA_R_, PC, PC_2UV_, and PET—under conditions of a microalgal photobioreactor illuminated with LEDs, in contact with culture medium and vapor. Thus, these results extend prior work documenting long-term PMMA reactor wall degradation [16].

Generally, it can be assumed that the reduction in light transmission through the witness samples is caused by (a) material degradation and (b) biofilm deposition on their surface.

It has been confirmed that all transparent polymers studied, after exposure to bioreactor conditions, exhibit significant degradation in terms of light transmission. The degree of light transparency loss is particularly high when measured for “as drained” samples. It has been further demonstrated that the reduction in light transmission is partially recoverable through cleaning with distilled water, which removes water-soluble biofilm. The permanent reduction in light transmission is the highest for PC and the lowest for PMMA_R_, amounting to 11.3% and 2.4%, respectively.

The greatest reduction in transmission between the virgin and after-cleaning samples, within the 450–700 nm range, was observed for PC (11.3%), while the smallest was recorded for PMMA_R_ (2.4%). Both materials are hydrophobic, promoting initial adsorption of protein and extracellular polymeric substances (EPS) that facilitate microbial adhesion [25,26,36].

PMMA is among the most widely used organic optical materials, valued for its high transparency and excellent optical properties. Its refractive index is sensitive to UV irradiation, which enables the fabrication of micro-structured elements such as gratings and waveguides. However, as reported by Rashidian and Dorranian (2014) [37], UV exposure also induces degradation of PMMA, involving chemical and structural changes that can compromise its optical performance. Our results confirm that PMMA samples sustained the lowest degradation of optical properties, out of polymers investigated, in conditions of the bioreactor.

Another widely used synthetic polymer valued for its low cost, durability, and high transparency [38,39] is polyethylene terephthalate (PET). Rostampour et al. (2024) [40] reported that UV radiation is the dominant factor driving PET degradation, leading to the reduction in the ester bond signal (1713 cm^−1^) and the appearance of a carbonyl band (1685 cm^−1^). Humidity and temperature were found to accelerate oxidative bond cleavage, although unexposed surfaces showed no detectable chemical changes. These findings highlight the sensitivity of PET to photo-oxidative processes and its limited stability under prolonged UV exposure. In turn, Redjala et al. (2020) [41] examined the effects of ultraviolet (UV) aging on polycarbonate (PC) using XRD, LM, SEM, microhardness, and tensile testing. UV exposure caused noticeable yellowing and a corresponding loss of transparency. For PET, UV radiation was identified as the dominant factor in chemical degradation, with humidity and temperature modulating the oxidative breakdown of ester bonds [40].

In our study, a reduction in light transmission was also observed for both PET and PC after exposure to bioreactor conditions. For PET, the decrease in transmission reached approximately 5%, while for PC it was markedly higher, at around 11%, despite the relatively short exposure period. These findings are consistent with the literature data, confirming the higher susceptibility of PC to environmental degradation processes compared to PET.

UV-protective additives, such as amorphous magnesium carbonate nanoparticles in PMMA, effectively reduce UV transmittance while preserving visible transparency, highlighting a potential strategy to enhance material longevity [42]. Taking this into account, the observed degradation patterns suggest that incorporating UV-protective additives (e.g., amorphous magnesium carbonate nanoparticles in PMMA) could further enhance the durability of transparent polymer walls in illuminated bioreactor environments.

Investigated in the present paper, PMMA and PMMA_R_ exhibited superior optical performance, maintaining high light transmission across the critical 400–700 nm range and demonstrating largely reversible changes in surface roughness and work of adhesion upon cleaning. In contrast, PC_2UV_ and PET showed pronounced optical degradation, particularly at the receding liquid interface, accompanied by permanent changes in resulting properties (light transmittance, surface roughness, and wettability). PC demonstrated intermediate behavior, with some irreversible changes that may promote biofilm formation.

The results presented in this paper emphasize differences in the surface degradation of the investigated materials in contact with vapor and with the culture medium. In the case of PMMA, we observe a clear dependence of the results on the zone in which the measurements were performed. The immersed areas exhibit less permanent degradation than those exposed to vapor, as indicated by the smaller decrease in transmission, the virtually unchanged roughness, and only a slight increase in work of adhesion compared to the vapor-exposed zone.

Analysis of the degradation process of the studied polymers (PMMA, PMMA_R_, PC, PC_2UV_, PET) during exposure in a bioreactor allowed us to quantify permanent and recoverable loss of transparency. Permanent loss, remaining after water cleaning, was observed to be accompanied by an increase in surface roughness (R_a_). According to the literature [43,44,45], PMMA should be more susceptible to micro-scratching and crazing, leading to localized roughness that accelerates biofilm nucleation in comparison to PC. Majrashi et al. (2024) [43] demonstrated that PC exhibits greater environmental and mechanical stability, especially under humid or alkaline conditions, maintaining smoother morphology over longer exposure periods. In our investigations, the effect of permanent degradation was most pronounced in submerged zones and areas with medium deposits, particularly for PC, PC_2UV_, and PET. In these materials, transparency loss was the highest, and the increase in post-cleaning R_a_ confirmed permanent surface changes, both in the submerged area and above the medium. In zone 5, representative of the submerged area, the R_a_ values were as follows:

PC—0.030 μm (an increase of 179% compared to the original material), PC_2UV_—0.040 μm (an increase of 379%), PET—0.023 μm (an increase of 110%), PMMA_R_—0.021 μm (an increase of 13%), and PMMA—0.017 μm (an increase of 11%). PMMA showed the lowest permanent degradation, especially in submerged zones, indicating better resistance to medium exposure. The transitional zone above the medium often exhibited higher transmission loss than the submerged zones, likely due to deposit accumulation and localized structural changes. The permanent transmission loss in this zone was 12.16% for PC and 6.83% for PMMA.

Recoverable degradation is mainly associated with surface properties and wettability, reflected in the contact angle and work of adhesion measurements. These changes were partially reversible after cleaning. PMMA and PMMA_R_ largely returned to baseline work of adhesion values, whereas PC_2UV_ and PET retained elevated work of adhesion in submerged or above-medium zones. The increase in work of adhesion is approximately 7% for PC_2UV_ and 5% for PET. This suggests that part of the degradation is superficial, likely due to medium adsorption or temporary polymer chain rearrangement.

Overall, this work demonstrates that PMMA and PMMA_R_ are recommended for transparent reactor walls to ensure sustained light delivery, whereas cleaning protocols should target regions most susceptible to biofilm formation, particularly at receding liquid zones.

## 5. Conclusions

This study provides a comparative evaluation of transparent polymers—PMMA, PMMA_R_, PC, PC_2UV_, and PET—under conditions simulating photobioreactor operation for microalgal cultivation. Prolonged exposure to culture medium and vapor significantly affected optical, roughness, and wetting properties, with material-dependent differences in susceptibility.

This study demonstrates that the type of polymer, zone of exposure, and contact with the culture environment strongly influence degradation mechanisms. Contact with vapor primarily affects transmission and roughness in transitional zones, with minor permanent damage. The cleaning process has little effect on these changes, indicating that degradation in this case is largely unaffected by rinsing. Contact with the culture medium induces both optical and surface modifications. Submerged zones show higher permanent degradation due to polymer–biofilm interaction, deposits, and bond breaking. Uncleaned samples retain biofilms, leading to more pronounced changes in light transmission and surface roughness. Cleaned samples reveal the underlying polymer degradation, which is correlated with roughness and partially affects wettability.

PMMA and PMMA_R_ demonstrated the highest optical stability and reversible surface modifications, confirming and extending previous findings on PMMA degradation behavior [16]. PC and PET showed moderate degradation, whereas PC_2UV_ exhibited the most pronounced loss of transmittance, particularly in immersed regions. Vapor-exposed zones generally experienced higher optical deterioration than fully immersed areas, indicating that biofilm formation at receding liquid interfaces is a key factor in transmission loss.

These results emphasize the importance of selecting polymers with stable optical and surface characteristics and optimizing reactor geometries and cleaning protocols to minimize partially wetted areas. Overall, the findings provide a foundation for predicting material lifetime, improving maintenance strategies, and guiding the design of durable, cost-effective transparent components in bioreactors and other water-contact systems.

From a practical perspective, the results suggest clear strategies to mitigate polymer degradation: minimizing exposure to vapor, reducing the extent of liquid contact, and applying careful rinsing after contact with aqueous suspensions. These measures help preserve optical and surface properties and limit permanent changes in polymer performance.

## Figures and Tables

**Table 1:** Manufacturer-provided properties of the investigated polymeric materials [https://tuplex.pl/ (accessed on 29 December 2025)].

Material	Trade Name	Transmittance [%]	Density [g × cm^−3^]
PC	MAKROLON^®^GP	88	1.20
PC_2UV_	MAKROLON^®^GP	87–91	1.20
PET	NUDEC^®^PET	88	1.27
PMMA	Altuglas^®^	92	1.19
PMMA_R_	Policril^®^Rec	90	1.19

**Table 2:** Results of surface roughness measurements for PC and PMMA samples measured after exposure and after cleaning, with differences relative to the virgin surface.

		*R_a_ After Exposure*	*R_a_ After Cleaning*	Difference	
				*After Exposure—Virgin*	*After Cleaning—Virgin*
	Zone	[μm]	[μm]	[μm]	[μm]
**PC**	**1**	0.034 ± 0.012	0.069 ± 0.044	0.027	0.062
	**2**	0.081 ± 0.037	0.028 ± 0.003	0.073	0.021
	**3**	0.018 ± 0.020	0.019 ± 0.002	0.010	0.012
	**4**	0.020 ± 0.007	0.019 ± 0.002	0.013	0.012
	**5**	0.020 ± 0.002	0.030 ± 0.002	0.013	0.023
	**6**	0.059 ± 0.037	0.041 ± 0.006	0.052	0.034
**PMMA**	**1**	0.010 ± 0.001	0.023 ± 0.002	−0.007	0.007
	**2**	0.006 ± 0.001	0.019 ± 0.001	−0.011	0.002
	**3**	2.006 ± 0.584	0.022 ± 0.016	1.989	0.005
	**4**	0.016 ± 0.001	0.017 ± 0.000	0.000	0.001
	**5**	0.018 ± 0.002	0.017 ± 0.001	0.002	0.001
	**6**	0.018 ± 0.001	0.018 ± 0.001	0.001	0.002

## Data Availability

The original contributions presented in this study are included in the article. Further inquiries can be directed to the corresponding authors.

## Abbreviations

The following abbreviations are used in this manuscript:

PMMA	poly(methyl methacrylate)
PMMA_R_	poly(methyl methacrylate) recycled
PC	polycarbonate
PC_2UV_	polycarbonate double UV layer
PET	polyethylene terephthalate
Ra	roughness average
ae	after exposure
ac	after cleaning
v	virgin

## References

[1] Nampoothiri K.M., Nair N.R., John R.P. (2010). An overview of the recent developments in polylactide (PLA) research. Bioresour. Technol..

[2] Moghbelli E., Banyay R., Sue H.-J. (2014). Effect of moisture exposure on scratch resistance of PMMA. Tribol. Int..

[3] Moharir R.V., Kumar S. (2019). Challenges associated with plastic waste disposal and allied microbial routes for its effective degradation: A comprehensive review. J. Clean. Prod..

[4] Wondraczek L., Bouchbinder E., Ehrlicher A., Mauro J.C., Sajzew R., Smedskjaer M.M. (2022). Advancing the Mechanical Performance of Glasses: Perspectives and Challenges. Adv. Mater..

[5] Speight J.G. (2020). Monomers, polymers, and plastics. Handbook of Industrial Hydrocarbon Processes.

[6] Beltrán-Sanahuja A., Casado-Coy N., Simó-Cabrera L., Sanz-Lázaro C. (2020). Monitoring polymer degradation under different conditions in the marine environment. Environ. Pollut..

[7] Yousif E., Haddad R. (2013). Photodegradation and photostabilization of polymers, especially polystyrene: Review. SpringerPlus.

[8] Andrady A.L. (2011). Microplastics in the marine environment. Mar. Pollut. Bull..

[9] Sero E.T., Siziba N., Bunhu T., Shoko R., Jonathan E. (2020). Biophotonics for improving algal photobioreactor performance: A review. Int. J. Energy Res..

[10] Olivieri G., Salatino P., Marzocchella A. (2014). Advances in photobioreactors for intensive microalgal production: Configurations, operating strategies and applications. J. Chem. Technol. Biotechnol..

[11] Posten C. (2012). Design and Performance Parameters of Photobioreactors. TATuP—Z. Tech. Theor. Und Prax..

[12] Muñoz R., Köllner C., Guieysse B. (2009). Biofilm photobioreactors for the treatment of industrial wastewaters. J. Hazard. Mater..

[13] Philip P., Jose E.T., Chacko J.K., Philip K., Thomas P. (2019). Preparation and characterisation of surface roughened PMMA electrospun nanofibers from PEO—PMMA polymer blend nanofibers. Polym. Test..

[14] Kiziltas E.E., Kiziltas A., Bollin S.C., Gardner D.J. (2015). Preparation and characterization of transparent PMMA–cellulose-based nanocomposites. Carbohydr. Polym..

[15] Kaddouri A., Serier B., Kaddouri K., Belhouari M. (2020). Experimental Analysis of the Physical Degradation of Polymers—The Case of Polymethyl Methacrylate. Fract. Struct. Integr..

[16] Borucinska E., Zamojski P., Grodzki W., Blaszczak U., Zglobicka I., Zielinski M., Kurzydlowski K.J. (2023). Degradation of Polymethylmethacrylate (PMMA) Bioreactors Used for Algal Cultivation. Materials.

[17] Serrano M.-A., Moreno J.C. (2020). Spectral transmission of solar radiation by plastic and glass materials. J. Photochem. Photobiol. B Biol..

[18] Wu X., Wang X., Qin Z., Zhang W. (2021). Polycarbonate composites with high light transmittance, haze, and flame retardancy based on a series of incomplete-cage oligomeric silsesquioxanes. J. Mater. Sci..

[19] Akbay I.K., Özdemir T. (2016). Monomer migration and degradation of polycarbonate via UV-C irradiation within aquatic and atmospheric environments. J. Macromol. Sci. Part A.

[20] Nair A., Sharma P., Sharma V., Diwan P. (2020). Effect of UV-irradiation on the optical properties of transparent PET polymeric foils. Mater. Today Proc..

[21] Santmarti A., Teh J.W., Lee K.-Y. (2019). Transparent Poly(methyl methacrylate) Composites Based on Bacterial Cellulose Nanofiber Networks with Improved Fracture Resistance and Impact Strength. ACS Omega.

[22] Moretti E., Zinzi M., Carnielo E., Merli F. (2017). Advanced Polycarbonate Transparent Systems with Aerogel: Preliminary Characterization of Optical and Thermal Properties. Energy Procedia.

[23] Avadanei M.I., Dimitriu D.G., Dorohoi D.O. (2024). Optical Anisotropy of Polyethylene Terephthalate Films Characterized by Spectral Means. Polymers.

[24] Guillard R.R.L. (1975). Culture of Phytoplankton for Feeding Marine Invertebrates. Culture of Marine Invertebrate Animals.

[25] Soriano-Jerez Y., Gallardo-Rodríguez J., López-Rosales L., García-Camacho F., Bressy C., Molina-Grima E., Cerón-García M. (2024). Preventing biofouling in microalgal photobioreactors. Bioresour. Technol..

[26] Cheah Y.T., Chan D.J.C. (2021). Physiology of microalgal biofilm: A review on prediction of adhesion on substrates. Bioengineered.

[27] He Y., Ji L., Yuan Y., Rui D., Li J., Cheng P., Sun L., Fan J. (2025). Recent advances in polysaccharide-dominated extracellular polymeric substances from microalgae: A review. Int. J. Biol. Macromol..

[28] Andac T., Weigmann P., Velu S.K.P., Pinçe E., Volpe G., Volpe G., Callegari A. (2019). Active matter alters the growth dynamics of coffee rings. Soft Matter.

[29] Callegari A., Andac T., Weigmann P., Velu S.K.P., Pince E., Volpe G., Volpe G. Active Matter Alters the Growth Dynamics of Coffee Rings. Proceedings of the Latin America Optics and Photonics Conference.

[30] Sempels W., De Dier R., Mizuno H., Hofkens J., Vermant J. (2013). Auto-production of biosurfactants reverses the coffee ring effect in a bacterial system. Nat. Commun..

[31] Gijsman P., Meijers G., Vitarelli G. (1999). Comparison of the UV-degradation chemistry of polypropylene, polyethylene, polyamide 6 and polybutylene terephthalate. Polym. Degrad. Stab..

[32] Çaykara T., Güven O. (1999). UV degradation of poly(methyl methacrylate) and its vinyltriethoxysilane containing copolymers. Polym. Degrad. Stab..

[33] Jabarin S.A. (1987). Crystallization kinetics of poly(ethylene terephthalate). III. Effect of moisture on the crystallization behavior of PET from the glassy state. J. Appl. Polym. Sci..

[34] Jördens C., Wietzke S., Scheller M., Koch M. (2010). Investigation of the water absorption in polyamide and wood plastic composite by terahertz time-domain spectroscopy. Polym. Test..

[35] Saccardo A., Bezzo F., Sforza E. (2022). Microalgae growth in ultra-thin steady-state continuous photobioreactors: Assessing self-shading effects. Front. Bioeng. Biotechnol..

[36] Costa J.A.V., Lucas B.F., Alvarenga A.G.P., Moreira J.B., De Morais M.G. (2021). Microalgae Polysaccharides: An Overview of Production, Characterization, and Potential Applications. Polysaccharides.

[37] Rashidian M., Dorranian D. (2014). Low-intensity UV effects on optical constants of PMMA film. J. Theor. Appl. Phys..

[38] Hopewell J., Dvorak R., Kosior E. (2009). Plastics recycling: Challenges and opportunities. Philos. Trans. R. Soc. B Biol. Sci..

[39] Nisticò R. (2020). Polyethylene terephthalate (PET) in the packaging industry. Polym. Test..

[40] Rostampour S., Cook R., Jhang S.-S., Li Y., Fan C., Sung L.-P. (2024). Changes in the Chemical Composition of Polyethylene Terephthalate under UV Radiation in Various Environmental Conditions. Polymers.

[41] Redjala S., Hocine N.A., Ferhoum R., Gratton M., Poirot N., Azem S. (2020). UV Aging Effects on Polycarbonate Properties. J. Fail. Anal. Prev..

[42] Yang J., Wang J., Strømme M., Welch K. (2021). Enhanced UV protection and water adsorption properties of transparent poly(methyl methacrylate) films through incorporation of amorphous magnesium carbonate nanoparticles. J. Polym. Res..

[43] Majrashi N.M., Al Qattan M.S., AlMubarak N.S., Alzahir K.Z., Gad M.M. (2024). Microbial Adhesion to Poly Methyl Methacrylate (PMMA) Denture Base Resins Containing Zinc Oxide (ZnO) Nanostructures: A Systematic Review of In Vitro Studies. Prosthesis.

[44] Hwang J.-J., Wu C.-Y., Hung Y.-H., Li M.-X., Luo K.-H., Jia H.-W., Balitaan J.N.I., Lin S.-R., Yeh J.-M. (2023). Biomimetic PMMA coating surface and its application on inhibition of bacterial attachment and anti-biofilm performance. Surf. Interfaces.

[45] Kavda S., Golfomitsou S., Richardson E. (2023). Effects of selected solvents on PMMA after prolonged exposure: Unilateral NMR and ATR—FTIR investigations. Herit. Sci..

